# 

**Published:** 1916-01-01

**Authors:** 


### CONTENTS.

PAGE

**The Nation's Awakening: an Opportunity for the Churches** ... ... ... ... ... ... 289

**Entertainments in Hospitals** ... ... ... 290

**Hospital and Institutional News** ... ... ... 291

Hospital Units in Serbia—Vague News of Various Hospitals—The Western Infirmary, Glasgow—Mr. Gilbert Barling's Retirement—The Red Cross Requisites Department—The Temporary Hospital Chapel—From Baby Clinic to Infants' Hospital—A Panel Doctor's Appeal to the Court—A Judge and Excessive Prescribing—The F.M.S. Hospital—The New Annexe at Blackburn Royal Infirmary—The Monthly Maintenance of Hospital Beds—Weather Protection in Open-Air Wards—A Confusion in Names—Supply of Drugs to a Military Hospital—Women as Anæsthetists—Potassium Chlorate Mistaken for Milk-Sugar—Poor-Law Officials and the Army—Educationists and Sir James Barr, M.D.—Committee Meetings in the Evening—The Army Council and the South Coast Hospitals—Guardians as Hospital Subscribers — An Executor's Right to Postpone Realisation—Monotony of Soliders in Hospital Dispelled—An Audacious Advertisement—Sanatorium Benefit Administration — British Lepers — This Week's Drug Market—To Our Readers.

**Christmas in the Hospitals** .. ... ... ... 297

England's Greeting.

PAGE

**Christmas Day at Guy's Hospital** ... ... 297

A Wonderful Atmosphere of Unselfish Effort.

**Christmas with the Wounded and Sick** ... 299

**Round the London Hospitals** ... . ... 299

**Some Military and Provincial Hospitals** ... 301

County of London War Hospital, Epsom—3rd London General Hospital, Wandsworth, S.W.—Out of the Limelight at Winchmore Hill—National Sanatorium, Benenden—Bradford—Addenbrooke's Hospital, Cambridge — 1st Eastern General Hospital, Cambridge—Leicester—Liverpool—Royal Victoria Infirmary, Newcastle—Norfolk and Norwich Hospital—Torquay.

**Round the World** ... ... ... ... ... 308

Fellow-Workers and the Roll of Honour.

**Parliament and the Profession** ... ... ... 309

**Passing Events and Topics** ... ... ... ... 309

**Personalia** ... ... ... ... ... ... 310

**Manchester and the Wounded** ... ... ... 310

**The Editor's Letter-Box** ... ... ... ... 310

**The Book Market** ... ... ... ... ... 310

**Institutional Needs** ... ... ... ... ... 310

**The Institutional Worker** ... ... (Suppl.) 1

The Feeding of Consumptives.

Question for January.

